# Metformin alleviates allergic airway inflammation and increases Treg cells in obese asthma

**DOI:** 10.1111/jcmm.16269

**Published:** 2021-01-09

**Authors:** Yimin Guo, Jianting Shi, Qiujie Wang, Luna Hong, Ming Chen, Shanying Liu, Xiaoqing Yuan, Shanping Jiang

**Affiliations:** ^1^ Department of Respiratory Medicine Sun Yat‐sen University Second University Hospital Sun Yat‐sen University Guangzhou China; ^2^ Guangdong Provincial Key Laboratory of Malignant Tumor Epigenetics and Gene Regulation Medical Research Center Sun Yat‐sen University Second University Hospital Sun Yat‐sen University Guangzhou China; ^3^ Institute of Pulmonary Diseases Sun Yat‐sen University Guangzhou China; ^4^ Research Center of Medicine Sun Yat‐sen University Second University Hospital Sun Yat‐sen University Guangzhou China; ^5^ Breast Tumor Center Sun Yat‐Sen Memorial Hospital Sun Yat‐Sen University Guangzhou China

**Keywords:** Metformin, Obese asthma, Tregs

## Abstract

Obesity increases the morbidity and severity of asthma, with poor sensitivity to corticosteroid treatment. Metformin has potential effects on improving asthma airway inflammation. Regulatory T cells (Tregs) play a key role in suppressing the immunoreaction to allergens. We built an obese asthmatic mouse model by administering a high‐fat diet (HFD) and ovalbumin (OVA) sensitization, with daily metformin treatment. We measured the body weight and airway inflammatory status by histological analysis, qRT‐PCR, and ELISA. The percentage of Tregs was measured by flow cytometry. Obese asthmatic mice displayed more severe airway inflammation and more significant changes in inflammatory cytokines. Metformin reversed the obese situation and alleviated the airway inflammation and remodelling with increased Tregs and related transcript factors. The anti‐inflammatory function of metformin may be mediated by increasing Tregs.

## 1 INTRO DUCTION

Asthma is one of the most frequent chronic respiratory diseases, which affects almost 25 million people around the world^1^; Obesity is a significant health problem and the prevalence of obesity was 42.4% in 2017.^2^ Obese asthma increases morbidity with worsening symptoms, with high frequencies and severe exacerbations and is insensitive to medical treatment.^3^ Therefore, an appropriate drug to treat obese asthma is urgently needed.

Metformin is regarded as a safe and curative medicine for reducing inflammation in several diseases, including asthma.^4^ A retrospective cohort study of the Taiwan National Health Insurance Research Database lasting 11 years indicated that metformin reduced the risk of asthma and diabetes mellitus (DM) compared to that in untreated patients.^4^ Metformin may be a potential therapeutic agent for treating obese asthma.

Tregs are important immune‐regulatory cells in the maintenance of immune tolerance. Tregs inhibit the activation and proliferation of effector T (Teff) cells by regulating and modulating the immune system which plays a key role in maintaining the immune tolerance of allergens. STAT5 regulates the Treg transcription factor Foxp3 and modulates the Tregs development. Tregs also activate mast cells, eosinophils, and basophils to suppress allergic inflammation and airway remodelling by activating IL‐6 or OX40/OX40L.^5^ Metformin‐elevated Tregs have been shown to alleviate inflammation and enhance the immunomodulating potential in autoimmune diseases. The therapeutic efficacy of metformin for treating obese asthma is scarce. Calixto found that metformin reduced airway eosinophilic inflammation in obese asthma.^6^ However, the changes in Tregs, a vital immune‐regulation call, have not been elucidated.

In the present study, we proved that metformin increased Tregs significantly and alleviated airway inflammation in obese asthma, which is more severe than non‐obese asthma.

## 2 MATERIALS AND METHODS

See Supplementary File.

## 3 RESULTS

### 3.1 Metformin treatment reduced body weight and alleviated the airway inflammation in obese asthma

**FIGURE 1:**
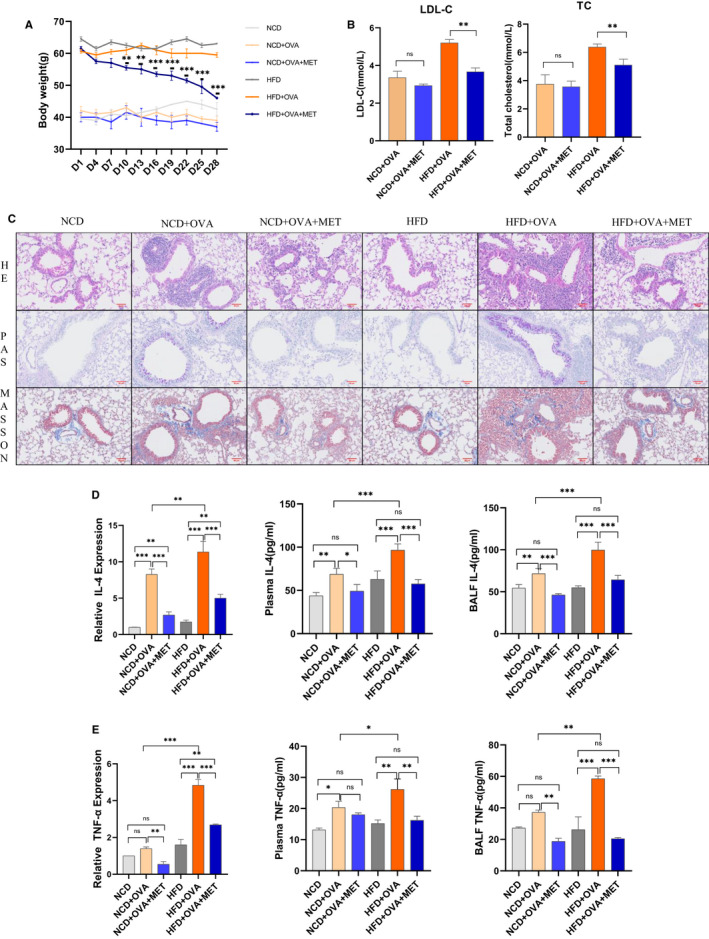
Metformin treatment reduced body weight and alleviated the airway inflammation in obese asthma. (A) Body weight changing curve of mice after treating with metformin. (B) Effect of metformin on the total cholesterol (TC) and low‐density lipoprotein cholesterol (LDL‐C) on mice of different treatment groups. (C) In the histological analysis, lung tissues were stained with H&E, Periodic Acid‐Schiff, and Masson’s trichrome staining to measure tissue inflammatory cells infiltration, mucus hyperproduction (red is positive for mucus), and collagen deposition (blue represent collagen deposition and red represent muscle fibre). (D, E) qRT‐PCR and ELISA analysis of inflammation cytokines IL‐4, TNF‐α in bronchoalveolar lavage fluid (BALF) cells, BALF, and plasma from different groups. All values are represented in the form of M ± SD (n = 5 ~ 8/group, *P *< 0.05 was statistically significant. ^*^ *P *< 0.05, ^**^ *P* < 0.01, and ^***^ *P* < 0.001 were compared with the indicated groups)

Mice fed with a high‐fat diet for 30 weeks exhibited a significant increase in their body weight, while the blood glucose showed non‐significant differences (Supplementary Figure S2A). Metformin treatment significantly decreased the body weight from the 10th day in obese asthmatic mice (Figure 1A), significantly lower the level of total cholesterol (TC) and low‐density lipoprotein cholesterol (LDL‐C), and elevated the level of high‐density lipoprotein cholesterol (HDL‐C) (Figure 1B, Supplementary Figure S2B).

The histological results indicated that the OVA‐challenged groups showed a significant increase in airway inflammation, except airway remodelling. Metformin treatment significantly alleviated airway inflammation and remodelling (Figure 1C, Supplementary Figure S2D). Inflammatory cytokines represent the inflammatory level. OVA stimulation increased the expressions of IL‐4 and TNF‐α and decreased the expressions of IL‐10 and IFN‐γ in the BALF and plasma, in obese asthma, while metformin reversed these changes (Figure 1D,E; Supplementary Figure S2E.F). It was concluded that metformin reduced the body weight and alleviated airway inflammation in obese asthma.

### 3.2 **Metformin treatment increased the percentage of Treg cells and Treg related transcript factors Foxp3 and STAT5**.

**FIGURE 2:**
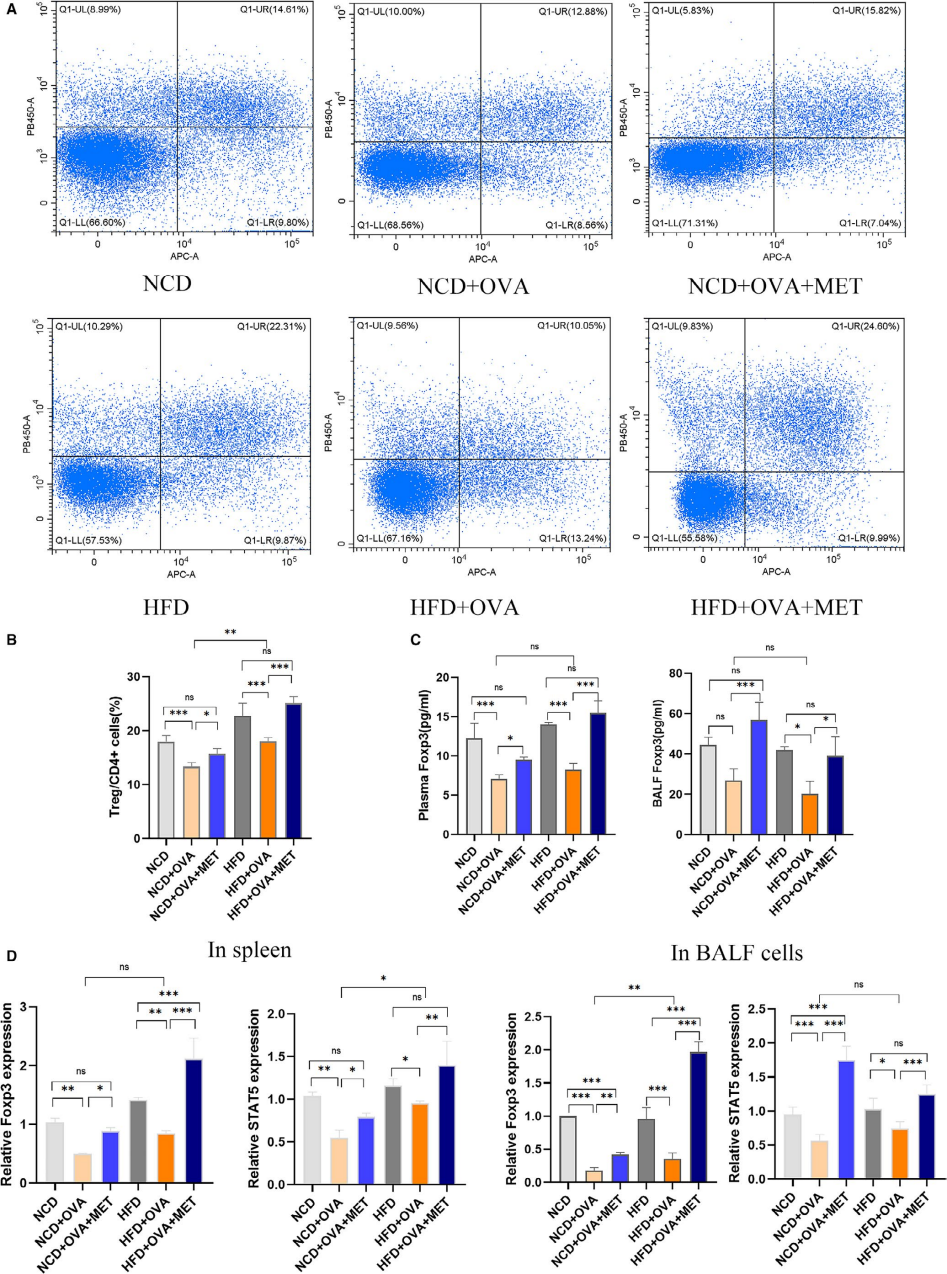
Effect of metformin on Treg cells and Treg related transcript factor Foxp3 and STAT5. (A) Flow cytometry analysis of Tregs (CD4^+^ Foxp3^+^) from the spleen of mice from different groups. (B) Quantitative calculation of Tregs from the total CD4^+^ cells in each group. (C) ELISA analysis of Treg related cytokine Foxp3 in BALF and plasma, from different groups of mice. (D) RT‐qPCR analysis of Treg related factors Foxp3 and STAT5 in BALF cells and spleen, from different groups of mice. All values are represented in the form of M ± SD (n = 5 ~ 8/group, *P *< 0.05 was statistically significant. ^*^ *P *< 0.05, ^**^ *P* < 0.01, and ^***^ *P *< 0.001 were compared with the indicated groups)

We calculated the percentage of Tregs in the spleen and found that the asthma groups (NCD + OVA group: 13.34 ± 0.77% and HFD + OVA group: 18.05 ± 0.67%) had a lower percentage of Tregs compared with non‐asthmatic groups (NCD group: 17.14 ± 1.15% and HFD group: 22.76 ± 2.39%). Metformin treatment (NCD + OVA + MET group: 15.68 ± 0.99% and HFD + OVA + MET group: 25.11 ± 1.27%) increased the percentage of Tregs compared with the non‐treated asthma group (NCD + OVA group: 13.34 ± 0.77% and HFD + OVA group: 18.05 ± 0.67%) (Figure 2A,B). OVA challenge reduced the percentage of Tregs with an increased expression of Foxp3 and STAT5; notably, metformin reversed this trend in obese asthma (Figure 2C,D).

Hence, metformin treatment increased the percentage of Tregs and its related transcript factors Foxp3 and STAT5, to provide a vital function in alleviating airway inflammation in obese asthma.

## 4 DISCUSSION

Obesity increases the severity and risk of asthma. Obese asthmatic patients are generally found to be unresponsive to ICS treatment, which greatly increases treatment difficulties.^3^ Metformin may be a potential treatment option because it not only reduces the weight and blood glucose, but also provides an anti‐inflammatory function in several diseases, including asthma.^4^ In the present study, we found that metformin alleviated the airway inflammation and remodelling with increased Tregs and its related transcript factors. The anti‐airway inflammation role of metformin may be mediated by increased Tregs.

Histological analyses demonstrated that obese asthma showed more severe airway inflammation, while airway remodelling was not significantly different. Additionally, metformin alleviated the airway inflammation. The levels of inflammatory cytokines IL‐4 and TNF‐α were increased while IL‐10 and IFN‐γ were decreased in obese asthma; metformin reversed these changes. IL‐4 was observed to participate in suppressing allergen‐specific Treg production and IgE antibody production. TNF‐α is produced by many immune‐related cells that down‐regulate Foxp3 expression, inhibit the function of Tregs, and play a major role in airway inflammation.^7^ IL‐10 is an effective inhibitor of monocyte/macrophage function and inhibits the production of several proinflammatory cytokines. The overexpression of IL‐4 and IL‐2 affected the expression of IL‐10 protein. Thymus Tregs promote the production of IL‐10, which in turn promotes peripheral Tregs production.^8^, ^9^ It was demonstrated that metformin alleviated obese asthma with severe airway inflammation, and Tregs played a vital role. Although IFN‐γ plays a proinflammatory role in diseases such as hematopoiesis,^10^ COPD, and asthma with influenza, it decreased in obese asthma. IFN‐γ inhibits the collection of eosinophils by inhibiting CD4+ T cell infiltration, reducing IL‐4 and IL‐5 production, inhibiting the synthesis of IgE, and attenuating allergy‐induced airway inflammation.^11^, ^12^

Tregs play a key role in inhibiting airway inflammation. Foxp3 is a specific Treg marker and STAT5 is a vital transcription factor that regulates Foxp3 and modulates Treg development. There were several STAT5 binding sites on the Foxp3 gene sequence. Tregs play a critical role in inhibiting airway inflammation. Lewkowich found additional severe airway inflammation and increased eosinophilic infiltration in the lungs after removing Tregs from the asthma model.^13^ Joetham injected antigen‐sensitized Tregs into asthmatic mice and found an effective reduction of airway inflammation.^14^ The regulatory function of metformin on Tregs has not yet been clarified in obese asthma. In some immune‐related inflammatory diseases, metformin inhibits the formation of the germ centre of the spleen and up‐regulates Tregs to inhibit the formation of Teff cells, inhibiting the production of autoantibodies^15^ to exert an anti‐inflammatory and anti‐autoimmune role. Hence, it is vital and necessary to explore the mechanism by which metformin plays an anti‐inflammatory role by increasing the Tregs in obese asthma.

In summary, we found that (a) obese asthmatic mice showed more severe airway inflammation; (b) metformin alleviated airway inflammation and increased Tregs in obese asthma. Metformin may be a therapeutic treatment for obese asthma and Tregs may be a therapeutic target.

## CONFLICT OF INTEREST

The authors claim they have no conflicts of interest.

## AUTHOR CONTRIBUTIONS

**Yimin Guo**: Conceptualization (equal); Data curation (equal); Formal analysis (equal); Investigation (equal); Methodology (equal); Resources (equal); Software (equal); Visualization (equal); Writing‐original draft (equal). **Jianting Shi**: Conceptualization (equal); Funding acquisition (equal); Project administration (equal); Supervision (equal). **Qiujie Wang**: Conceptualization (equal); Data curation (equal); Formal analysis (equal); Investigation (equal). **Luna Hong**: Formal analysis (equal); Methodology (equal); Software (equal); Validation (equal). **Ming Chen**: Methodology (equal); Software (equal). **Shanying Liu**: Conceptualization (equal); Funding acquisition (equal); Project administration (equal); Visualization (equal); Writing‐review & editing (equal). **Xiaoqing Yuan**: Funding acquisition (lead); Project administration (lead); Supervision (lead); Visualization (lead); Writing‐review & editing (lead). **Shanping Jiang**: Funding acquisition (lead); Project administration (lead); Supervision (lead); Validation (lead); Writing‐review & editing (lead).

## Supporting information

Supplementary Material1

Supplementary Material2

## Data Availability

The data that support the findings of this study are openly available in [repository name e.g “figshare”] at http://doi.org/[doi], reference number [reference number].
