# Supplementary material for: Metformin alleviates allergic airway inflammation and increases Treg cells in obese asthma
Source: J Cell Mol Med. 2021 Jan 9;25(4):2279–84. doi: 10.1111/jcmm.16269 (PMC7882927; doi:10.1111/jcmm.16269)

Supplementary Figure 1:

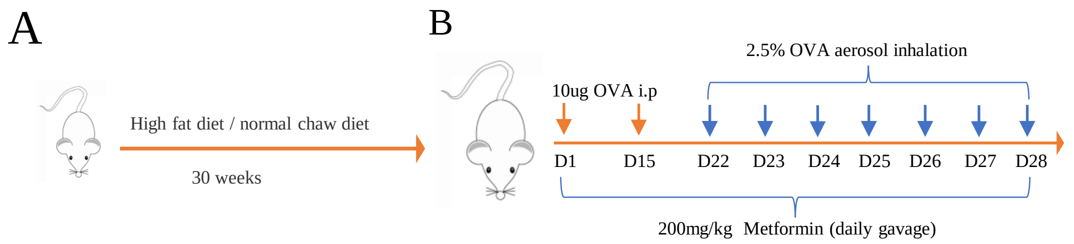

Supplementary Figure 1: Experimental design of ovalbumin induced obesity asthmatic mice and metformin treatment.

Supplementary Table 1: Primer sequences applied for gene expression analyses in BALF cells and spleen .

Gene	Forward primer 5'-3'	Reverse primer 5'-3'
IL-4	GGTCTCAACCCCCAGCTAGT	GCCGATGATCTCTCTCAAGTGAT
IL-10	GCTGGACAACATACTGCTAACC	ATTTCCGATAAGGCTTGGCAA
TNF-α	CAGGCGGTGCCTATGTCTC	CGATCACCCCGAAGTTCAGTAG
INF-γ	ACAGCAAGGCGAAAAAGGATG	TGGTGGACCACTCGGATGA
Foxp3	ACCATTGGTTTACTCGCATGT	TCCACTCGCACAAAGCACTT
STAT5	CTGGGCATGTCCCGAGATG	GAGGGGTCTTGACCACTGG
β-actin	GAAATCGTGCGTGACATCAAAG	TGTAGTTTCATGGATGCCACAG

Supplementary Figure 2:

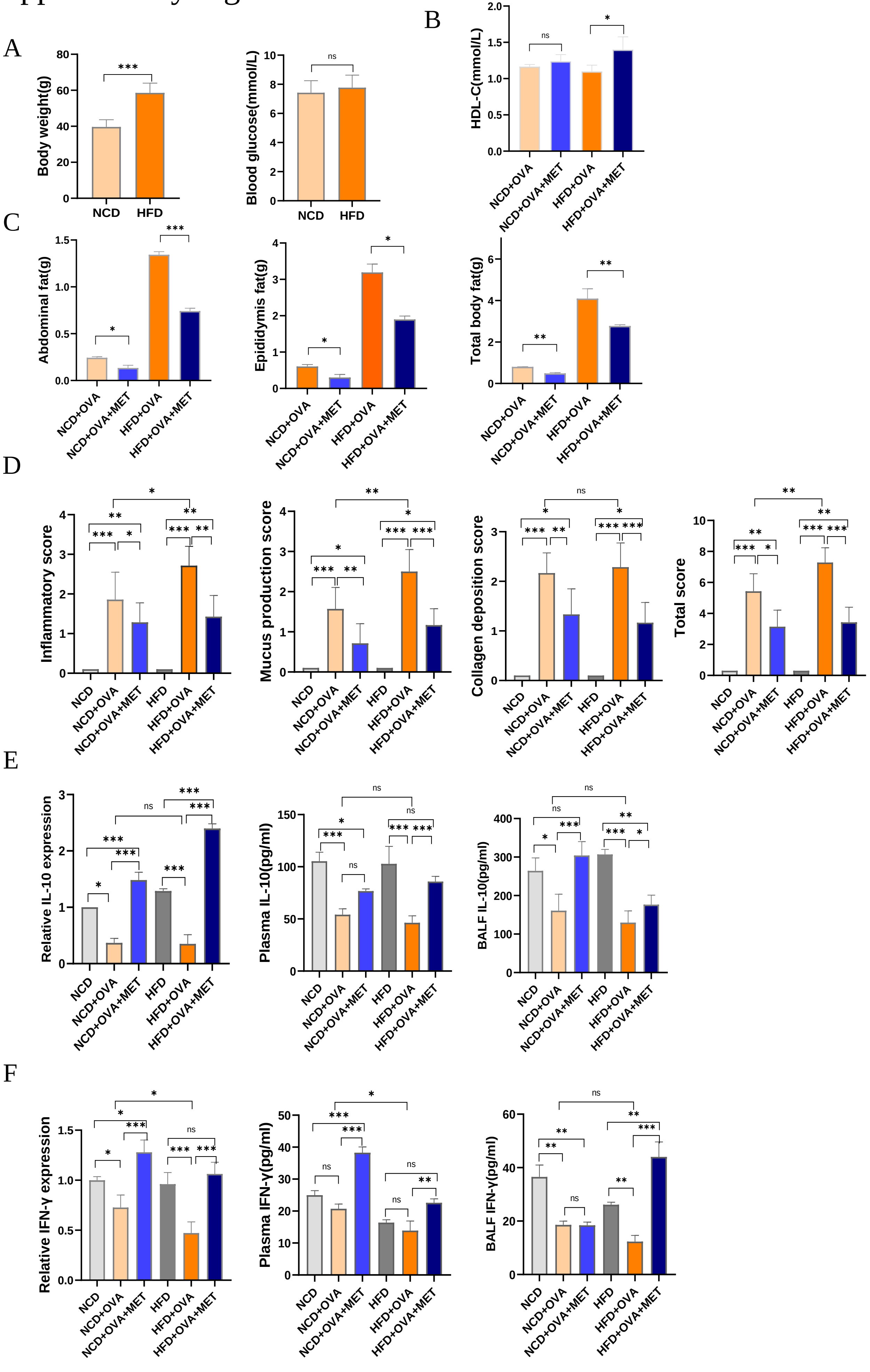

Supplement: Supplementary file 1 — Supplementary Material1 [file JCMM-25-2279-s001.pdf]
