# Supplementary material for: Metformin alleviates allergic airway inflammation and increases Treg cells in obese asthma
Source: J Cell Mol Med. 2021 Jan 9;25(4):2279–84. doi: 10.1111/jcmm.16269 (PMC7882927; doi:10.1111/jcmm.16269)
Supplement: Supplementary file 2 — Supplementary Material2 [file JCMM-25-2279-s002.docx]

**2. Materials and methods:**

**2.1 Animal and diet**

Male CD1 mice were provided by Dr. Tailin Li bought from Sun Yat-sen university animal experimental center (Guangzhou, china). Four mice per cage had a constant temperature (22-25c) and 12 hours of light-dark cycle, and fed with a normal chaw diet (70% of the protein, 20% of the protein, 10% of the fat) or a high fat diet (35% of the carbohydrate, 20% of the protein, 45% of the fat) in a 12-hour light-dark cycle (RDI, USA) (Supplementary Figure 1A). Body weight was measured every three days and blood glucose were measured once a week. All animal experimental procedures and protocols are based on the ethics of laboratory research at Sun Yat-sen University and approved by the Center of Animal Experiment of Sun Yat-sen University (2020000444).

**2.2 OVA-stimulated asthmatic mice model and metformin treatment.**

Mice were divided into six groups according to the random number table and each group containing eight mice: normal chaw diet group (NCD group); non-obese asthma group (NCD+OVA group); metformin treatment non-obese asthma group (NCD+OVA+MET group); high fat diet group (HFD group); obese asthma group (HFD+OVA group); metformin treatment obese asthma group (HFD+OVA+MET group). In the first and the 14^th^ days, the mouse abdominal injection 0.2ml normal saline contains 10ug ovalbumin (Sigma-Aldrich, St. Louis) and10 mg Al(OH)_3_ gel. From days 21 to 28, mice were inhaled 30 minutes of ovalbumin aerosol (2.5%) solution in a sealed container everyday (supplementary figure 1b). Aerosols were generated with an atomizer (OMRON, japan) and delivered to the sealed container. Non-sensitized mice groups (NCD group and HFD group) were used normal saline in the same way as a control group. The metformin treatment groups received 200mg/kg/day metformin (Sigma Aldrich, USA) by gavage from the first OVA sensitization. Non-treatment groups (NCD groups and HFD groups) are treated with normal saline in the same way as controls. (Supplementary Figure 1B).

**2.3 Samples collection**

Within 24h after the last challenge, intraperitoneally injection of 1% pentobarbital sodium (50mg/kg) to anaesthetize mice. Body weight, epididymal fat and peritoneally fat were measured after sacrificing. Blood samples were collected by removing eyeball and stored in EDTA anticoagulation tubes in 4℃. Samples were centrifuged at 3000g for 15min to get the supernatant and stored at -80℃. Immediately after blood collection, tying off the right bronchus to remove the right lung and fixed in the 4% paraformaldehyde. Cannulating the trachea and using 5ml ice-cold PBS to lavage the left lung to get the bronchoalveolar lavage fluid (BALF). The first 1.5ml BALF was used to measure cytokines by ELISA. BALF cells were collected by centrifuging at 1000 rpm at temperature for 10 minutes. Added 1ml RBC lysis to get rid of RBC at room temperature for 10min and then centrifuged at 1000 rpm at room temperature for 10 minutes to get BALF cells. Soak the right lung in the 4% formaldehyde for at least 24 hours, then change 70% ice ethanol until bury it in a paraffin. Cut it into a thin piece approximately 4 mm.

**2.4 Histological analysis**

Inflammatory cells infiltrating status were assessed semi-quantitatively by HE staining according to the following scoring roles: no inflammatory cells infiltrate (score 0); a few inflammatory cells infiltrate (<25% ) (score 1); focal inflammatory cells infiltrate (25-75%) (score 2); almost completely inflammatory cell infiltrate (75-100%) (score 3). Goblet cells mucus production was assessed by PAS staining using the following scoring roles: no red staining (score 0); red staining involving <25% of bronchus (score 1); red staining involving approximately 25-75% of bronchus (score 2); red staining involving approximately 75-100% of bronchus (score 3). Collagen deposition status were assessed by Masson’s trichrome staining using the following scoring roles (The blue indicating the positive tatus for collagen deposition): no parabronchial blue staining (score 0); blue staining involving <25% of bronchus (score 1); blue staining involving approximately 25-75% of bronchus (score 2); blue staining involving approximately 75-100% of bronchus (score 3). The total score was added up by the above three scores. Slides were examined using Olympus microscope (magnification, ×200).

**2.5 Measurement of plasma biochemical indexes**

The blood glucose level was measured by the glucometer (Roche, Switzerland). The level of total cholesterol (TC), low density lipoprotein cholesterol (LDL-C) and high density lipoprotein cholesterol (HDL-C) in plasma were determined by biochemical analyzer (Servicebio, Wuhan, china)

**2.6 ELISA analysis of BALF samples.**

Plasma and BALF were collected to measure the concentration of IL-4 , IL-10, TNF-α, IFN-γ and FOXP3 with commercially available enzyme-linked immunosorbent assay (ELISA) kits(IL-4 and IL-10, BD biosciences, Franklin lakes, NJ, USA; TNF-α, IFN-γ and FOXP3, geneTex, Alton Pkwy Irvine, CA, USA) according to the manufacturer’s protocol. Using Multi-function enzyme spectrometer (TECAN Spark10M, TECAN, Switzerland) to detect the absorbance at 450nm.

**2.7qRT-PCR**

BALF cells and spleen cells were isolated using TRIzol (TAKARA, Japan). The concentration of RNA was detected by NanoDrop One spectrophotometer (Thermo,USA). Reverse transcription of 500ng RNA by using the Superscript Reverse Transcription System (Takara, Shiga, Japan) to become cDNA. Quantitative real-time PCR (qRT-PCR) of cDNA was performed with DNA master SYBR green II (Takara, Shiga, Japan) by using a Roche 480 PCR machine. Primer sequences are available in Supplementary Table 1.

**2.8 Flow cytometry analysis**

The spleen was crushed into a single-cell suspension. Using red blood cells (RBC) lysis buffer to get rid of RBC and centrifugate at 3000 rpm at 4℃ for 15 min to get the single cells. Spleen cells were stained with fluorescence-labeled antibodies specific for FITC anti-mouse CD4 (Biolegend, USA), Brilliant Violet 421™ anti-mouse FOXP3 (Biolegend, USA) and Fixable Viability Dye eFluor™ 450 (eBioscience, USA). Subsequently, the cells were tested by using cytoExp software (ThermoFisher, USA).

**2.9 Statistical analysis**

Statistical analyses were performed using SPSS 20.0 software. Differences among treatment groups were analyzed using the one-way ANOVA. Differences of body weight and glucose between normal chow diet mice and high fat diet mice were analyzed using student’s unpaired *t*-test. All data were expressed as the means ± standard deviation (SD). A *p*-value<0.05 and the representative is statistically significant.

**Supplementary figure legend:**

**Supplementary Figure 1**: Experimental design of ovalbumin induced obesity asthmatic mice and metformin treatment.

**Supplementary Figure 2**: Metformin treatment reduced body weight and alleviated the airway inflammation in obese asthma.

(A) Body weight and blood glucose of mice fed with different diet before metformin treatment.

(B) Effect of metformin on the high-density lipoprotein cholesterol (HDL-C) on mice of different treatment groups

(C) Effect of metformin on abdominal and epididymis fat weight from mice of different treatment groups.

(D) Inflammation infiltration, mucus production and collagen deposition were measured by the inflammation score, goblet hyperplasia score and fibrotic score for each group. Total score was measured by adding above scores for each group.

(E, F) qRT-PCR and ELISA analysis of inflammation cytokines IL-10, IFN-γ in bronchoalveolar lavage fluid (BALF) cells, BALF, and plasma from different groups.

All values are represented in the form of M ± SD (n=5~8/group, p<0.05 was statistically significant.* p<0.05, ** p <0.01, and *** p <0.001 were compared with the indicated groups).
